# Estimation of upper and lower bounds of Gini coefficient by fuzzy data

**DOI:** 10.1016/j.dib.2020.105288

**Published:** 2020-02-14

**Authors:** Reza Ashraf Ganjoei, Hossein Akbarifard, Mashaallah Mashinchi, Sayyed Abdol Majid Jalaee Esfandabadi

**Affiliations:** a Department of Economics, Faculty of Management and Economics, Shahid Bahonar University of Kerman, Kerman, Iran; b Department of Statistics, Faculty of Mathematics and Computer, Shahid Bahonar University of Kerman, Kerman, Iran

**Keywords:** Fuzzy data, Gini coefficient, Asymmetric coefficients, Uncertainty

## Abstract

The data presented in this paper are used to examine the uncertainty in macroeconomic variables and their impact on the Gini coefficient. Annual data for the period 2017 - 1996 are taken from the Bank of Iran website https://www.cbi.ir. We used fuzzy regression with symmetric coefficients to calculate upper and lower bound data of Gini coefficient. Estimated data at this stage can be a very useful guide for policymakers, on the other hand, it is a benchmark for evaluating the effectiveness of government policies. The reason for using fuzzy regression to estimate data on Gini coefficients is the extra flexibility of this model.

**Table:** Specifications Table

Subject	Economics and Fuzzy Logic
Specific subject area	These data are related to the uncertainties in macroeconomic policies that affect the Gini coefficient.
Type of data	Table; Graph; Figure
How data were acquired	Data on economic variables were obtained from the Central Bank of Iran and then, using Gaussian software and fuzzy regression, the Gini coefficients of upper and lower and middle bounds were calculated.
Data format	Raw
Parameters for data collection	Data on variables of economic instability in Iran
Description of data collection	Data on the independent variable including five variables inflation, exchange rate, bank interest rate, GDP and stock price index and related variable data (Gini coefficient) were collected from the Central Bank of Iran website.
Data source location	IRAN
Data accessibility	https://data.mendeley.com/datasets/nr8cptwgf8/draft?a=40f49de2-34d5-4bb1-bc98-5c59c5f02163

**Table:** 

**Value of the Data** • This data is a very useful guide for policymakers and governments as it provides a measure of the effectiveness or inefficiency of their policies. • This data is used by the Planning and Budgeting Organization, the Tax and Holding Authority, the Central Bank and other subsidiary organizations. • Using fuzzy logic and fuzzy regression to predict, we can analyze the future income distribution trend. • The efficiency of government policies is determined by comparing the upper, middle and lower Gini coefficient data with the current Gini coefficient data.

## 1 Data description

**Fig. 1:**
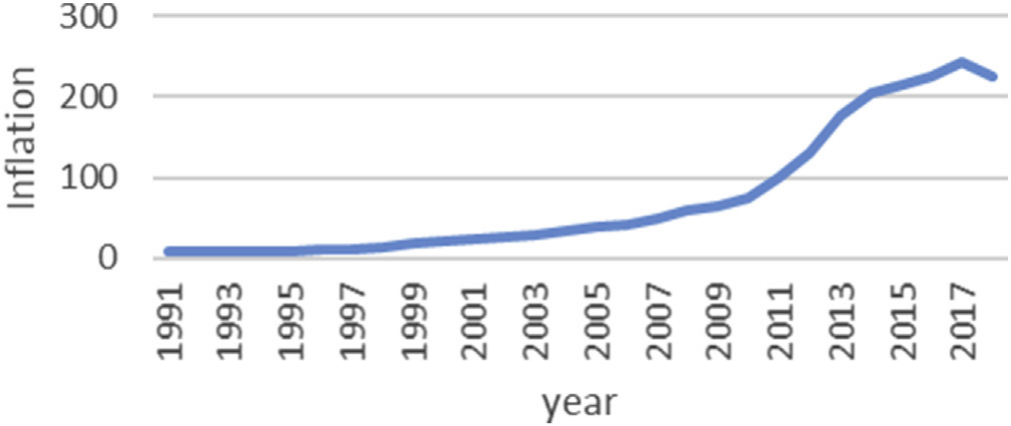
Graph inflation.

**Fig. 2:**
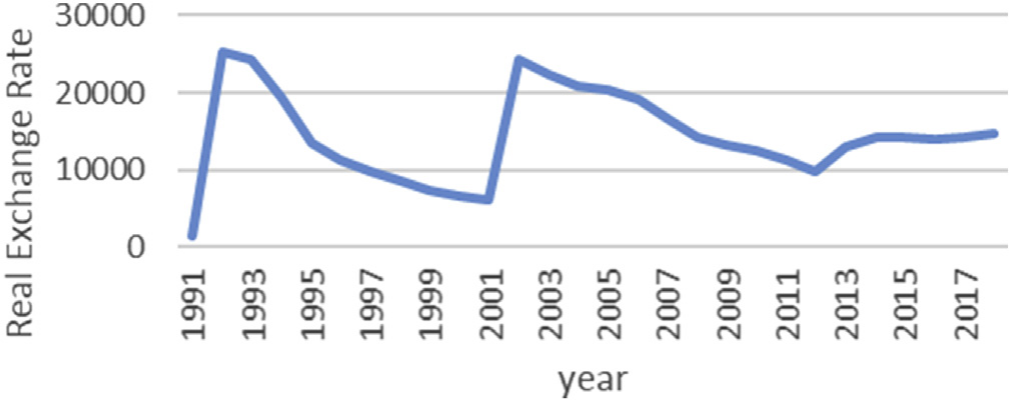
Graph Real exchange rates.

**Fig. 3:**
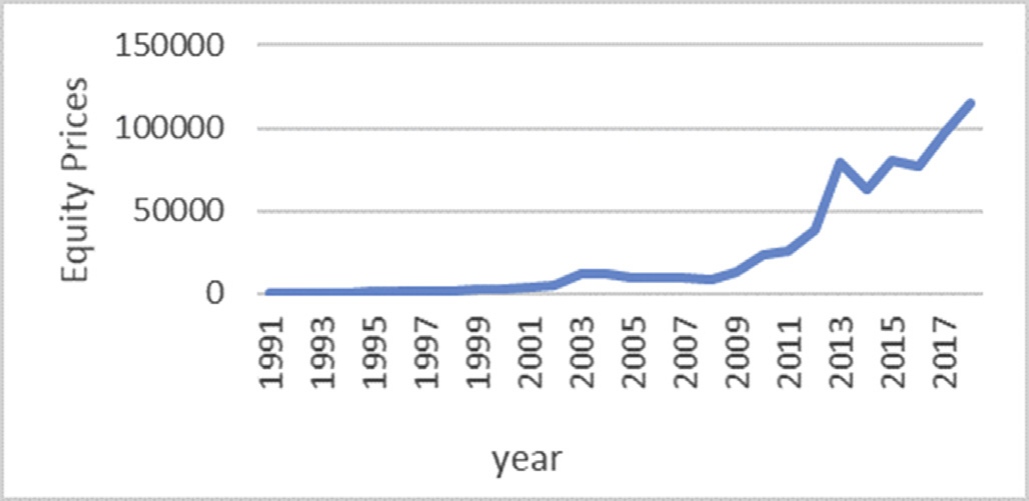
Graph stock price index.

**Fig. 4:**
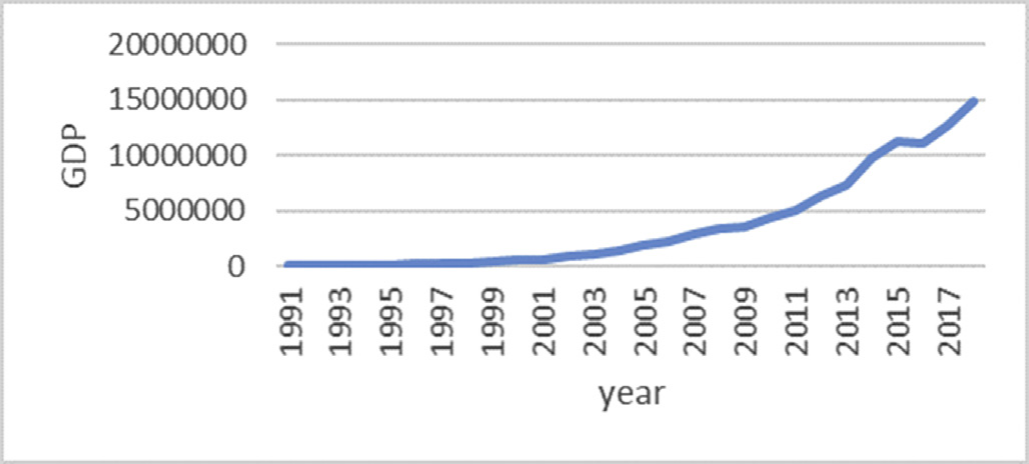
Graph GDP.

**Fig. 5:**
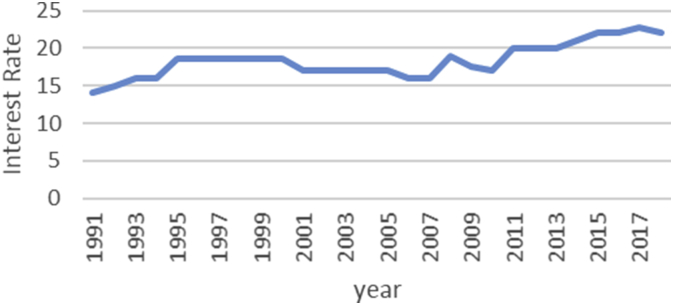
Graph bank interest rate.

**Fig. 6:**
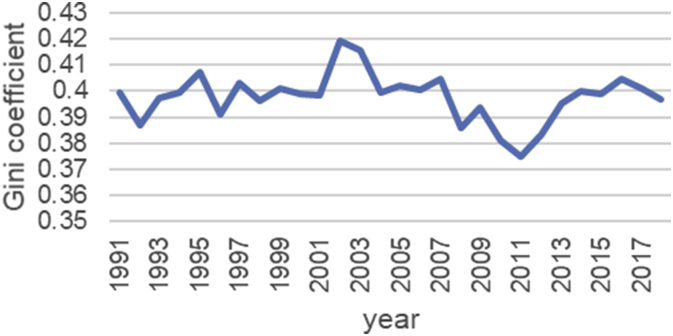
Graph Gini coefficient.

The Gini coefficient is one of the most important indicators introduced by Corrado Gini to measure inequality, which is always nonnegative and has values between zero and one [1,2]. A combination of macroeconomic variables including GDP, bank interest rates, inflation rate, exchange rate and stock price index, an index called economic policy uncertainty (EPU) is introduced [[1], [2]]. For estimate the upper and lower bound data, the Gini coefficient of EPU index data from 1991 to 2018 is taken from the Bank of Iran website https://www.cbi.ir. Also, all data is available in the Mendeley (https://data.mendeley.com/datasets/nr8cptwgf8/draft?a=40f49de2-34d5-4bb1-bc98-5c59c5f02163). As can be seen in Fig. 1, inflation has been rising over time. Although it has been slow in some years, the trend of inflation is generally rising. Fig. 2 shows the trend of the real exchange rate fluctuation in different years. Fig. 3, Fig. 4, respectively, show that GDP and stock price index have fluctuated over time, although they have fluctuated in some years. Fig. 5 also shows the fluctuating movement of bank interest rates. Fig. 6 shows the trend of the Gini coefficient of movement in different years. The fluctuations of this variable over time indicate that the instability of economic variables that will have a major impact on the Gini coefficient, depending on government policies.

## 2 Experimental design, materials, and method

In this section, the theoretical foundations of the regression model with symmetric and asymmetric fuzzy coefficients are briefly stated [[3], [4], [5]], where the general form of the regression model with fuzzy coefficients is as in Eq. (1) in order to compare the results of logistic smooth transition autoregressive model to the linear regression model with symmetric and asymmetric fuzzy coefficients.

(1) $$\tilde{\text{Y}}=\text{f}\left(\underline{\text{x}},\text{A}\right)={\tilde{\text{A}}}_{0}+{\tilde{\text{A}}}_{1}{\text{x}}_{1}+{\tilde{\text{A}}}_{2}{\text{x}}_{2}+\ldots +{\tilde{\text{A}}}_{\text{n}}{\text{x}}_{\text{n }}$$

here $\tilde{\;Y}$ is the dependent variable or fuzzy output, $x=\left(x_{1},x_{2},\ldots x_{n}\right)$ is the vector of independent variables or input vector, $A=\left\{{\tilde{A}}_{0},{\tilde{A}}_{1},\ldots .{\tilde{A}}_{n}\right\}$ is a set of fuzzy numbers, and $\left(y_{1},x_{1}\right),\left(y_{2},x_{2}\right)\ldots ..,\left(y_{m},x_{m}\right)$ are the a set of regular data. The fuzzy parameters ${\tilde{\;A}}_{0},{\tilde{A}}_{1},\ldots .{\tilde{A}}_{n}$ are meant to be determined in a way to have the best fitting on the above data in accordance with Eq. (1) based on some of the goodness-of-fit criteria. So, the general form of the membership function. $\tilde{A\;}$ can be written as Eq. (2) with respect to three parameters as the center a, low width $s^{L}$ and right width $s^{R}$ [3]:

(2) $$\tilde{A}\left(\text{x}\right)=\{\begin{array}{c} 1-\frac{a-x}{s^{L}}\;\;\;a-s^{L}\leq x\leq a\;\;\\ 1-\frac{x-a}{s^{R}}\;\;a<x\leq a+s^{R}\;\; \end{array}$$

This membership function can also be displayed in another way. That is, the high width is expressed based on a low width. Thus, k $\;s^{L}$ = $s^{R}$ is placed in the above membership function, in which k is a real and positive number known as the kurtosis coefficients [3]. Therefore, the asymmetric triangular fuzzy number $\tilde{A}$ can also be described by ${\tilde{A}=\left(a,s^{L},k\right)}_{\text{T}}$. In this case, the membership function $\tilde{A}$ is represented by Eq. (3)

(3) $$\tilde{\text{A}}\left(\text{x}\right)=\{\begin{array}{c} 1-\frac{\text{a}-\text{x}}{{\text{s}}^{\text{L}}}\;\;\;a-{\text{s}}^{\text{L}}\leq x\leq a\\ 1-\frac{\text{x}-\text{a}}{{\text{ks}}^{\text{R}}}\;\;a<x\leq a+k{\text{s}}^{\text{R}} \end{array}$$

### 2.1 Model with symmetric coefficients

If ${\tilde{\;A}}_{i}$s are fuzzy symmetric numbers  i=0,1,2,…,n, and $x_{i}$ s are the real and positive numbers, then, according to Eq. (1), the fuzzy output $\tilde{\;Y}$ will be a symmetric triangular fuzzy number ${\tilde{Y}=(\;f^{c}\left(\underline{\text{x}}\right),f^{s}\left(\underline{\text{x}}\right))}_{\text{T}}$, in which $f^{c}\left(\underline{\text{x}}\right)$ is the center and $f^{s}\left(\underline{\text{x}}\right)$ is the width of $\;\tilde{Y}$, which are obtained by Eq. (4) and Eq. (5) as below [5].

(4) $$f^{c}\left(\underline{x}\right)=a_{0}+a_{1}x_{1}+\ldots +a_{n}x_{n}\;$$

(5) $$f^{s}\left(\underline{x}\right)=s_{0}+s_{1}x_{1}+\ldots +s_{n}x_{n}$$

In other words, the fuzzy output $\tilde{\text{Y}}$ membership function is:

(6) $$\tilde{\;Y}\left(y\right)=\{\begin{array}{c} 1-\frac{f^{c}\left(\underline{x}\right)-y}{f^{s}\left(\underline{x}\right)}\;\;f^{c}\left(\underline{x}\right)-f^{s}\left(\underline{x}\right)\leq y\leq \;f^{c}\left(\underline{x}\right)\;\\ 1-\frac{y-f^{c}\left(\underline{x}\right)}{f^{s}\left(\underline{x}\right)}\;\;f^{c}\left(\underline{x}\right)<y\leq \;f^{c}\left(\underline{x}\right)+f^{s}\left(\underline{x}\right)\; \end{array}$$

### 2.2 Estimating the parameters of fuzzy regression model

For estimate the parameters of the fuzzy regression model Eq. (2), We consider two criterias. First, the membership value of each $y_{i}$ in ${\tilde{\;Y}}_{i}$ should be a large number. In this case, it is ensured that the fuzzy model has a good fitting to the observations [3]. Thus, we are looking for a model that: 1) fuzzy output, $\tilde{\text{Y}}$ for all values $\;{\tilde{Y}}_{j}$ , has a membership degree as large as h, that is:

(7) $${\tilde{Y}}_{j}\left(y_{j}\right)\geq h\;,\;i=1,2\ldots .,m$$

2) The fuzzy coefficients ${\tilde{A}}_{i}$, are such that ambiguity of the fuzzy output ${\tilde{Y}}_{j}\;$ is minimized.

In the case where ${\tilde{\;A}}_{i}$s are symmetric, the total fuzzy output width $\tilde{\text{ Y}}$ for all data is called the objective function. The Objective function for the case where ${\tilde{\;A}}_{i}\text{s}$ are asymmetric is represented by Eq. (6).

(8) $$Z=2ms_{0}+2\overset{n}{\underset{i=1}{\sum }}\left(s_{i}\overset{m}{\underset{j=1}{\sum }}x_{ji}\right)$$

In Eq. (8), $x_{ji}$ means the jth observation of the ith variable. In the case where ${\tilde{\;A}}_{i}$ s are asymmetric, objective function [3] is represented by Eq. (9) [3].

(9) $$Z=m\left(s_{0}^{L}+s_{0}^{R}\right)+\overset{n}{\underset{i=1}{\sum }}\left[\left(s_{0}^{L}+s_{0}^{R}\right)\overset{m}{\underset{j=1}{\sum }}x_{ji})\right]$$

The objective function Z, by substituting $k_{i}s_{i}^{L}$ = $s_{i}^{R}$, can be written based on kurtosis coefficients as follows.

(10) $$Z=\left(1+K_{0}\right)ms_{0}^{L}+\overset{n}{\underset{i=1}{\sum }}\left[\left(\left(1+K_{i}\right)s_{i}^{L}\right)\overset{m}{\underset{j=1}{\sum }}x_{ji})\right]\;$$

### 2.3 FLR Model with symmetric coefficients

If ${\tilde{\;A}}_{i}$ in Eq. (2) are considered as symmetric with regard to Eq. (7) and Eq. (9) then the constraints Eq. (11) and Eq. (12) are obtained [3] as follows

(11) $$\left(1-h\right)s_{0}+\left(1-h\right)\overset{n}{\underset{i=1}{\sum }}\left(s_{0}x_{ji}\right)-a_{0}-\overset{n}{\underset{i=1}{\sum }}\left(s_{0}x_{ji}\right)\geq -y_{i\;},\;j=1,2,\ldots .,m\;$$

(12) $$\left(1-h\right)s_{0}+\left(1-h\right)\overset{n}{\underset{i=1}{\sum }}\left(s_{0}x_{ji}\right)+a_{0}+\overset{n}{\underset{i=1}{\sum }}\left(s_{0}x_{ji}\right)\geq +y_{i\;},\;j=1,2,\ldots .,m\;$$

**Fig. 7:**
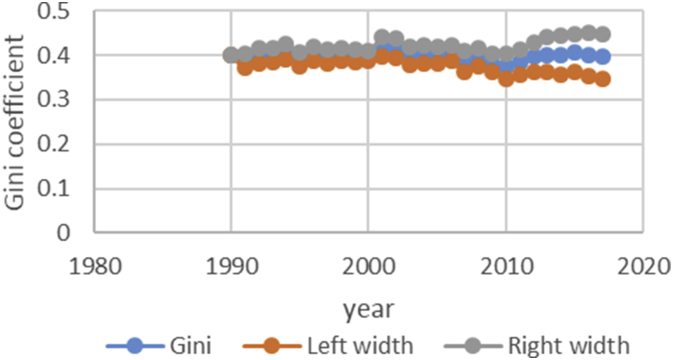
Right and left widths Gini coefficient for degree of membership 0.1.

**Fig. 8:**
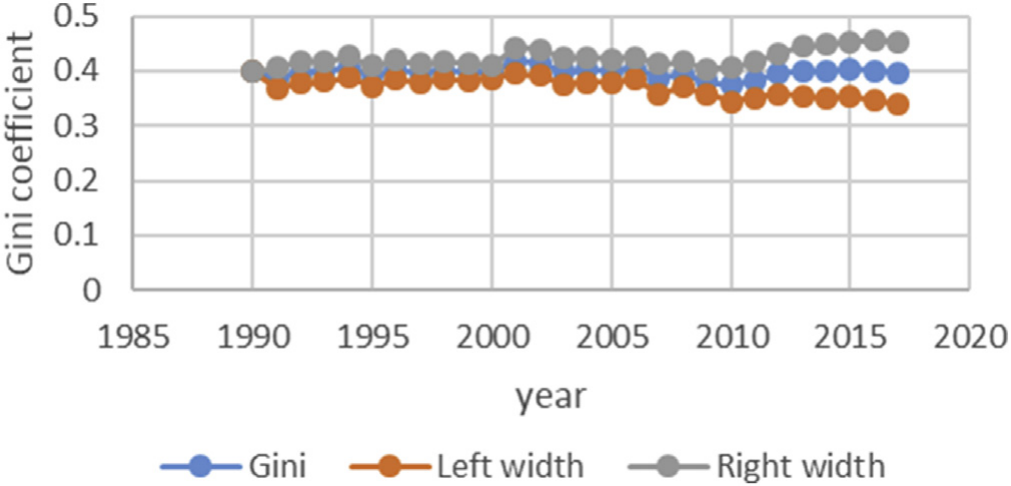
Right and left widths Gini coefficient for degree of membership 0.2.

**Fig. 9:**
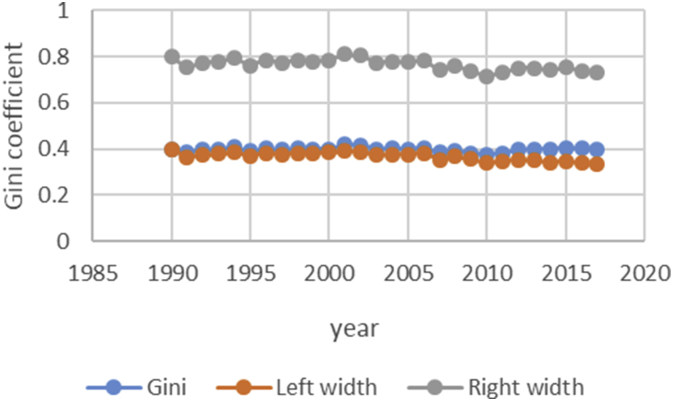
Right and left widths Gini coefficient for degree of membership 0.3.

**Fig. 10:**
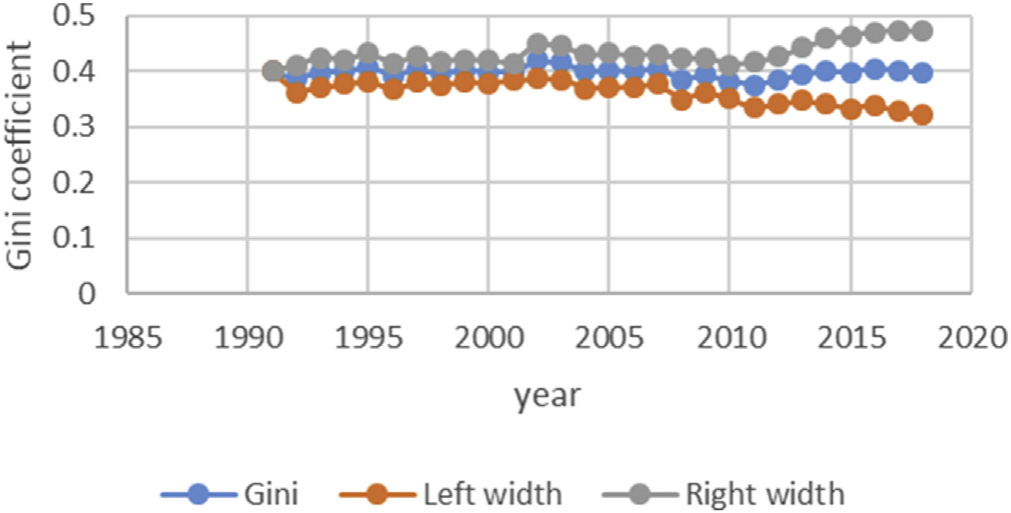
Right and left widths Gini coefficient for degree of membership 0.4.

**Fig. 11:**
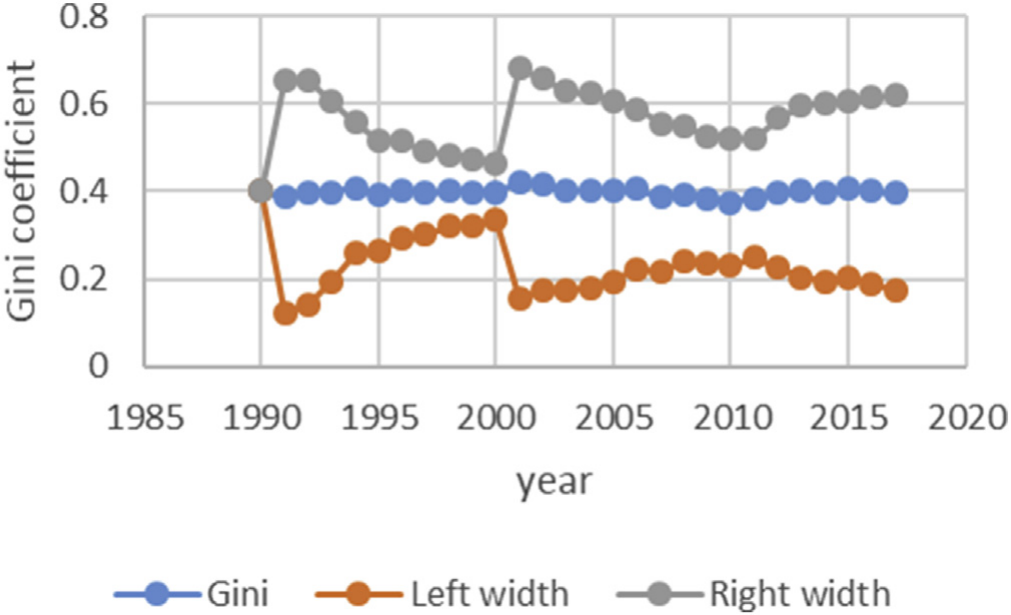
Right and left widths Gini coefficient for degree of membership 0.5.

**Fig. 12:**
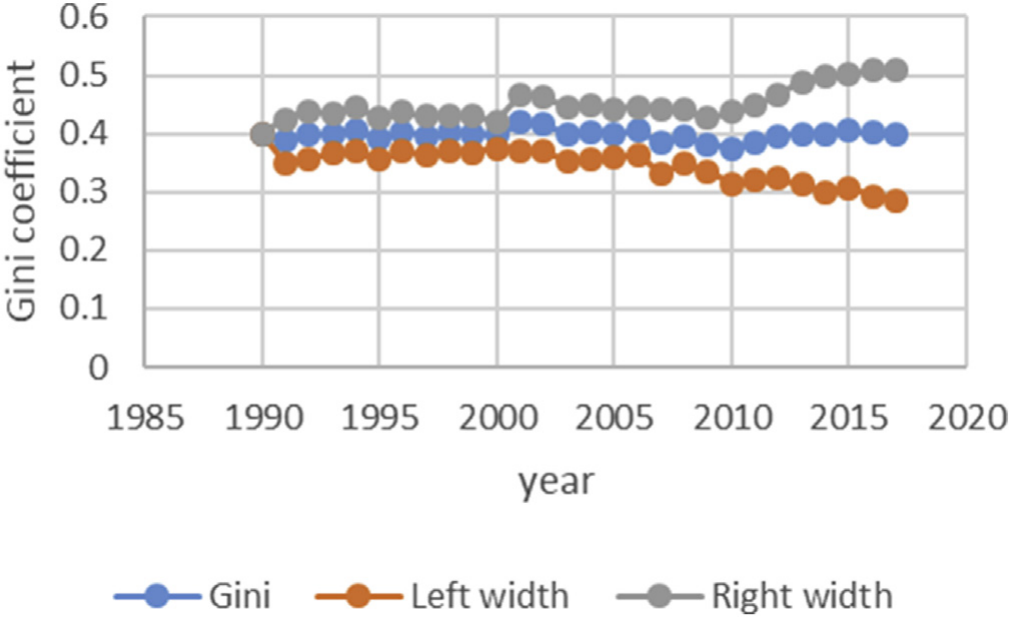
Right and left widths Gini coefficient for degree of membership 0.6.

**Fig. 13:**
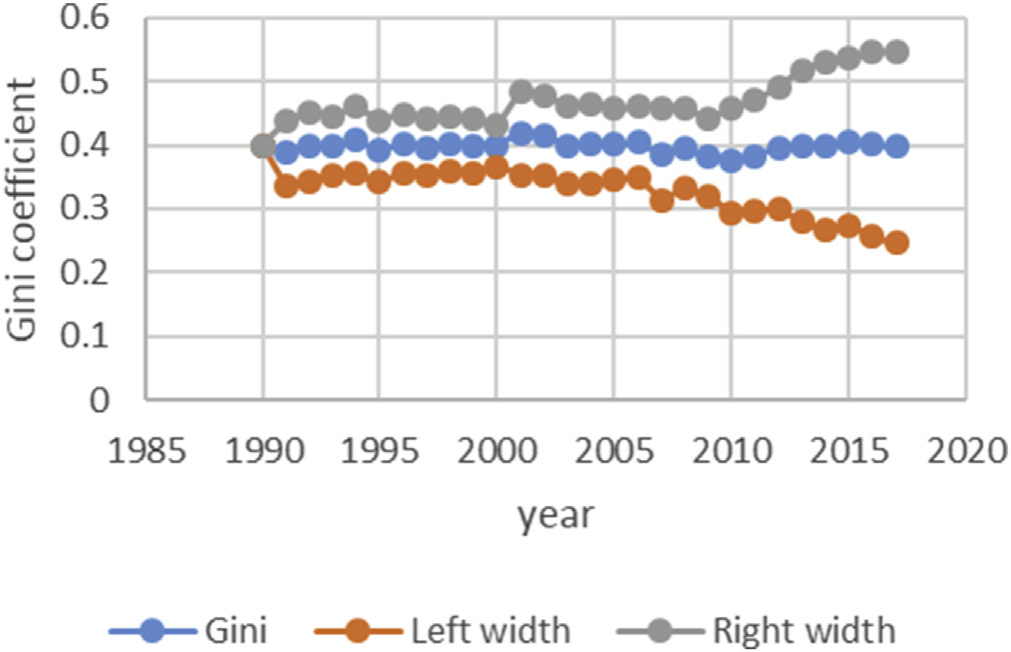
Right and left widths Gini coefficient for degree of membership 0.7.

**Fig. 14:**
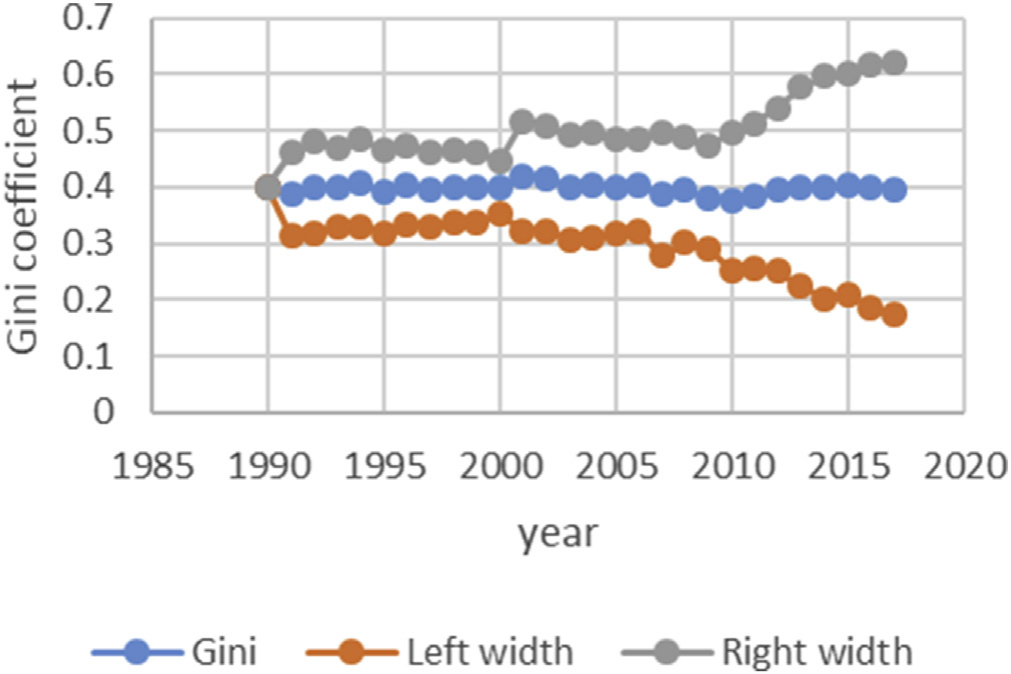
Right and left widths Gini coefficient for degree of membership 0.8.

**Fig. 15:**
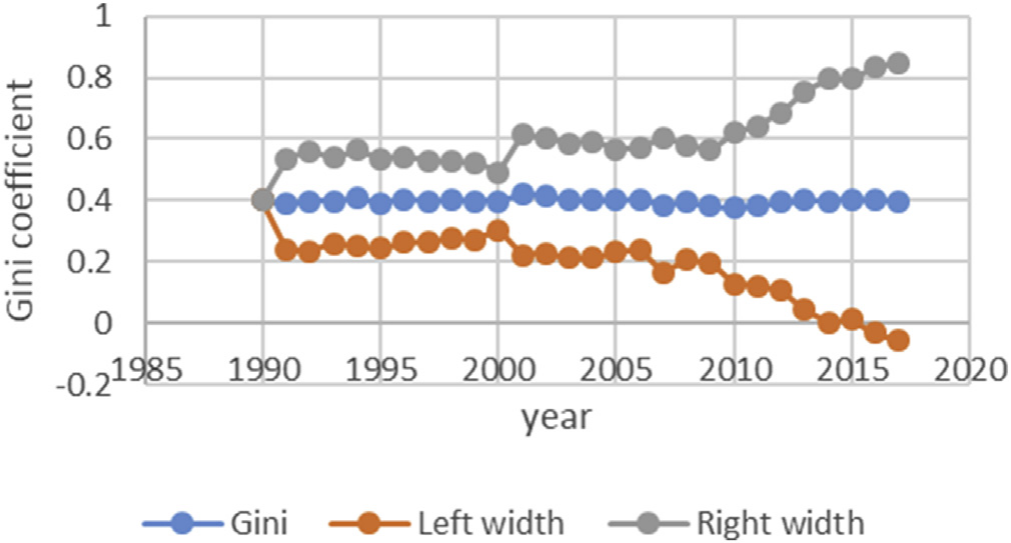
Right and left widths Gini coefficient for degree of membership 0.9.

After describing the method of calculating the upper and lower bound data Gini coefficient, we have programmed the data of variables related to the EPU attribute using fuzzy regression using GAMS software and calculated the optimal probabilities for the relevant variables. Next, we calculate the upper and lower bound data of the Gini coefficient for the membership degree of 0.1–09. The data are presented in Table 1, Table 2, Table 3, Table 4, Table 5. Fig. 7, Fig. 8, Fig. 9, Fig. 10, Fig. 11, Fig. 12, Fig. 13, Fig. 14, Fig. 15.

**Table 1:** Calculation of the right and left widths of the Gini coefficient for membership degrees of 0.1 and 0.2.

Year	h = 0.1			h = 0.2		
	Width	Left width	Right width	Width	Left width	Right width

1991	0	0.3996	0.3996	0	0.3996	0.3996
1992	0.016199	0.370801	0.403199	0.018545	0.368455	0.405545
1993	0.017828	0.379772	0.415428	0.020438	0.377162	0.418038
1994	0.015057	0.384243	0.414357	0.01727	0.38203	0.41657
1995	0.017038	0.390362	0.424438	0.019613	0.387787	0.427013
1996	0.01587	0.37513	0.40687	0.018276	0.372724	0.409276
1997	0.015122	0.387778	0.418022	0.01742	0.38548	0.42032
1998	0.014368	0.382132	0.410868	0.016556	0.379944	0.413056
1999	0.013813	0.387087	0.414713	0.015918	0.384982	0.416818
2000	0.013698	0.385402	0.412798	0.015782	0.383318	0.414882
2001	0.010293	0.388207	0.408793	0.011837	0.386663	0.410337
2002	0.021313	0.397787	0.440413	0.024422	0.394678	0.443522
2003	0.020558	0.395042	0.436158	0.023551	0.392049	0.439151
2004	0.0203	0.3793	0.4199	0.023244	0.376356	0.422844
2005	0.020616	0.381684	0.422916	0.023589	0.378711	0.425889
2006	0.018405	0.381995	0.418805	0.021013	0.379387	0.421413
2007	0.018075	0.386425	0.422575	0.020614	0.383886	0.425114
2008	0.024032	0.361868	0.409932	0.027499	0.358401	0.413399
2009	0.02052	0.37338	0.41442	0.023429	0.370471	0.417329
2010	0.020241	0.361059	0.401541	0.023065	0.358235	0.404365
2011	0.027184	0.347816	0.402184	0.031072	0.343928	0.406072
2012	0.028684	0.354716	0.412084	0.032733	0.350667	0.416133
2013	0.032004	0.363196	0.427204	0.036492	0.358708	0.431692
2014	0.039243	0.360657	0.439143	0.044699	0.355201	0.444599
2015	0.043798	0.355002	0.442598	0.049882	0.348918	0.448682
2016	0.043458	0.361142	0.448058	0.049499	0.355101	0.454099
2017	0.048016	0.352784	0.448816	0.054671	0.346129	0.455471
2018	0.050172	0.346828	0.447172	0.05703	0.33997	0.45403

**Table 2:** Calculation of the right and left widths of the Gini coefficient for membership degrees of 0.3 and 0.4.

Year	h = 0.3			h = 0.4		
	Width	Left width	Right width	Width	Left width	Right width

1991	0	0.3996	0.7992	0	0.3996	0.3996
1992	0.020892	0.366108	0.753108	0.024355	0.362645	0.411355
1993	0.023052	0.374548	0.772148	0.026855	0.370745	0.424455
1994	0.019488	0.379812	0.779112	0.022696	0.376604	0.421996
1995	0.022198	0.385202	0.792602	0.025809	0.381591	0.433209
1996	0.020696	0.370304	0.761304	0.024055	0.366945	0.415055
1997	0.019735	0.383165	0.786065	0.022933	0.379967	0.425833
1998	0.018764	0.377736	0.774236	0.021799	0.374701	0.418299
1999	0.01805	0.38285	0.78375	0.020963	0.379937	0.421863
2000	0.017901	0.381199	0.780299	0.020786	0.378314	0.419886
2001	0.013423	0.385077	0.783577	0.015589	0.382911	0.414089
2002	0.02759	0.39151	0.81061	0.032111	0.386989	0.451211
2003	0.026617	0.388983	0.804583	0.030972	0.384628	0.446572
2004	0.026283	0.373317	0.772917	0.030574	0.369026	0.430174
2005	0.026684	0.375616	0.777916	0.031034	0.371266	0.433334
2006	0.023772	0.376628	0.777028	0.027646	0.372754	0.428046
2007	0.023343	0.381157	0.785657	0.027132	0.377368	0.431632
2008	0.031192	0.354708	0.740608	0.036221	0.349679	0.422121
2009	0.026577	0.367323	0.761223	0.030861	0.363039	0.424761
2010	0.026178	0.355122	0.736422	0.030388	0.350912	0.411688
2011	0.035295	0.339705	0.714705	0.040951	0.334049	0.415951
2012	0.03721	0.34619	0.72959	0.043154	0.340246	0.426554
2013	0.04147	0.35373	0.74893	0.048104	0.347096	0.443304
2014	0.050818	0.349082	0.748982	0.058933	0.340967	0.458833
2015	0.056726	0.342074	0.740874	0.065774	0.333026	0.464574
2016	0.05629	0.34831	0.75291	0.065269	0.339331	0.469869
2017	0.062184	0.338616	0.739416	0.072095	0.328705	0.472895
2018	0.064887	0.332113	0.729113	0.075216	0.321784	0.472216

**Table 3:** Calculation of the right and left widths of the Gini coefficient for membership degrees of 0.5 and 0.6.

Year	h = 0.5			h = 0.6		
	Width	Left width	Right width	Width	Left width	Right width

1991	0	0.3996	0.3996	0	0.3996	0.3996
1992	0.264048	0.122952	0.651048	0.036974	0.350026	0.423974
1993	0.25697	0.14063	0.65457	0.040645	0.356955	0.438245
1994	0.204595	0.194705	0.603895	0.034305	0.364995	0.433605
1995	0.148964	0.258436	0.556364	0.038702	0.368698	0.446102
1996	0.125547	0.265453	0.516547	0.036025	0.354975	0.427025
1997	0.110446	0.292454	0.513346	0.03431	0.36859	0.43721
1998	0.095386	0.301114	0.491886	0.03258	0.36392	0.42908
1999	0.082064	0.318836	0.482964	0.031296	0.369604	0.432196
2000	0.075887	0.323213	0.474987	0.031013	0.368087	0.430113
2001	0.064619	0.333881	0.463119	0.023289	0.375211	0.421789
2002	0.262183	0.156917	0.681283	0.048442	0.370658	0.467542
2003	0.242176	0.173424	0.657776	0.046686	0.368914	0.462286
2004	0.227954	0.171646	0.627554	0.04605	0.35355	0.44565
2005	0.222447	0.179853	0.624747	0.046713	0.355587	0.449013
2006	0.206152	0.194248	0.606552	0.041649	0.358751	0.442049
2007	0.182905	0.221595	0.587405	0.040808	0.363692	0.445308
2008	0.169834	0.216066	0.555734	0.054201	0.331699	0.440101
2009	0.153875	0.240025	0.547775	0.046221	0.347679	0.440121
2010	0.145722	0.235578	0.527022	0.045499	0.335801	0.426799
2011	0.146151	0.228849	0.521151	0.061123	0.313877	0.436123
2012	0.134874	0.248526	0.518274	0.064351	0.319049	0.447751
2013	0.170356	0.224844	0.565556	0.071808	0.323392	0.467008
2014	0.195609	0.204291	0.595509	0.087934	0.311966	0.487834
2015	0.203503	0.195297	0.602303	0.09809	0.30071	0.49689
2016	0.200922	0.203678	0.605522	0.097332	0.307268	0.501932
2017	0.212941	0.187859	0.613741	0.107483	0.293317	0.508283
2018	0.220873	0.176127	0.617873	0.112145	0.284855	0.509145

**Table 4:** Calculation of the right and left widths of the Gini coefficient for membership degrees of 0.7 and 0.8.

Year	h = 0.7			h = 0.8		
	Width	Left width	Right width	Width	Left width	Right width

1991	0	0.3996	0.3996	0	0.3996	0.3996
1992	0.049709	0.337291	0.436709	0.074063	0.312937	0.461063
1993	0.054667	0.342933	0.452267	0.081518	0.316082	0.479118
1994	0.046148	0.353152	0.445448	0.068838	0.330462	0.468138
1995	0.05212	0.35528	0.45952	0.077919	0.329481	0.485319
1996	0.048522	0.342478	0.439522	0.072564	0.318436	0.463564
1997	0.046218	0.356682	0.449118	0.069134	0.333766	0.472034
1998	0.043893	0.352607	0.440393	0.065674	0.330826	0.462174
1999	0.042169	0.358731	0.443069	0.063106	0.337794	0.464006
2000	0.04179	0.35731	0.44089	0.06254	0.33656	0.46164
2001	0.031374	0.367126	0.429874	0.046921	0.351579	0.445421
2002	0.065175	0.353925	0.484275	0.097227	0.321873	0.516327
2003	0.062816	0.352784	0.478416	0.093715	0.321885	0.509315
2004	0.061963	0.337637	0.461563	0.092442	0.307158	0.492042
2005	0.062856	0.339444	0.465156	0.093768	0.308532	0.496068
2006	0.056029	0.344371	0.456429	0.083526	0.316874	0.483926
2007	0.054904	0.349596	0.459404	0.081845	0.322655	0.486345
2008	0.072979	0.312921	0.458879	0.108974	0.276926	0.494874
2009	0.062219	0.331681	0.456119	0.092842	0.301058	0.486742
2010	0.061242	0.320058	0.442542	0.091341	0.289959	0.472641
2011	0.082316	0.292684	0.457316	0.122932	0.252068	0.497932
2012	0.086662	0.296738	0.470062	0.129389	0.254011	0.512789
2013	0.096688	0.298512	0.491888	0.144302	0.250898	0.539502
2014	0.118399	0.281501	0.518299	0.176669	0.223231	0.576569
2015	0.13208	0.26672	0.53088	0.197094	0.201706	0.595894
2016	0.13106	0.27354	0.53566	0.195578	0.209022	0.600178
2017	0.144729	0.256071	0.545529	0.215965	0.184835	0.616765
2018	0.150988	0.246012	0.547988	0.225204	0.171796	0.622204

**Table 5:** Calculation of the right and left widths of the Gini coefficient for membership degrees of 0.9.

Year	h = 0.9		
	Width	Left width	Right width

1991	0	0.3996	0.3996
1992	0.149126	0.237874	0.536126
1993	0.163996	0.233604	0.561596
1994	0.138438	0.260862	0.537738
1995	0.15635	0.25105	0.56375
1996	0.145552	0.245448	0.536552
1997	0.138637	0.264263	0.541537
1998	0.131661	0.264839	0.528161
1999	0.12648	0.27442	0.52738
2000	0.125333	0.273767	0.524433
2001	0.09408	0.30442	0.49258
2002	0.195465	0.223635	0.614565
2003	0.188376	0.227224	0.603976
2004	0.185795	0.213805	0.585395
2005	0.188447	0.213853	0.590747
2006	0.167938	0.232462	0.568338
2007	0.16452	0.23998	0.56902
2008	0.218712	0.167188	0.604612
2009	0.186419	0.207481	0.580319
2010	0.183437	0.197863	0.564737
2011	0.246612	0.128388	0.621612
2012	0.259559	0.123841	0.642959
2013	0.289574	0.105626	0.684774
2014	0.354533	0.045367	0.754433
2015	0.39548	0.00332	0.79428
2016	0.39243	0.01217	0.79703
2017	0.433328	0.032528	0.834128
2018	0.451965	0.054965	0.848965

## Ethics approval

Ethics approval is not applicable.

## Consent for publication

The authors of the research have given their consent for the data to be used and published in this scientific article.
